# Supplementary figures and images for: The utilization of advance telemetry to investigate critical physiological parameters including electroencephalography in cynomolgus macaques following aerosol challenge with eastern equine encephalitis virus
Source: PLoS Negl Trop Dis. 2021 Jun 17;15(6):e0009424. doi: 10.1371/journal.pntd.0009424 (PMC8259972; doi:10.1371/journal.pntd.0009424)

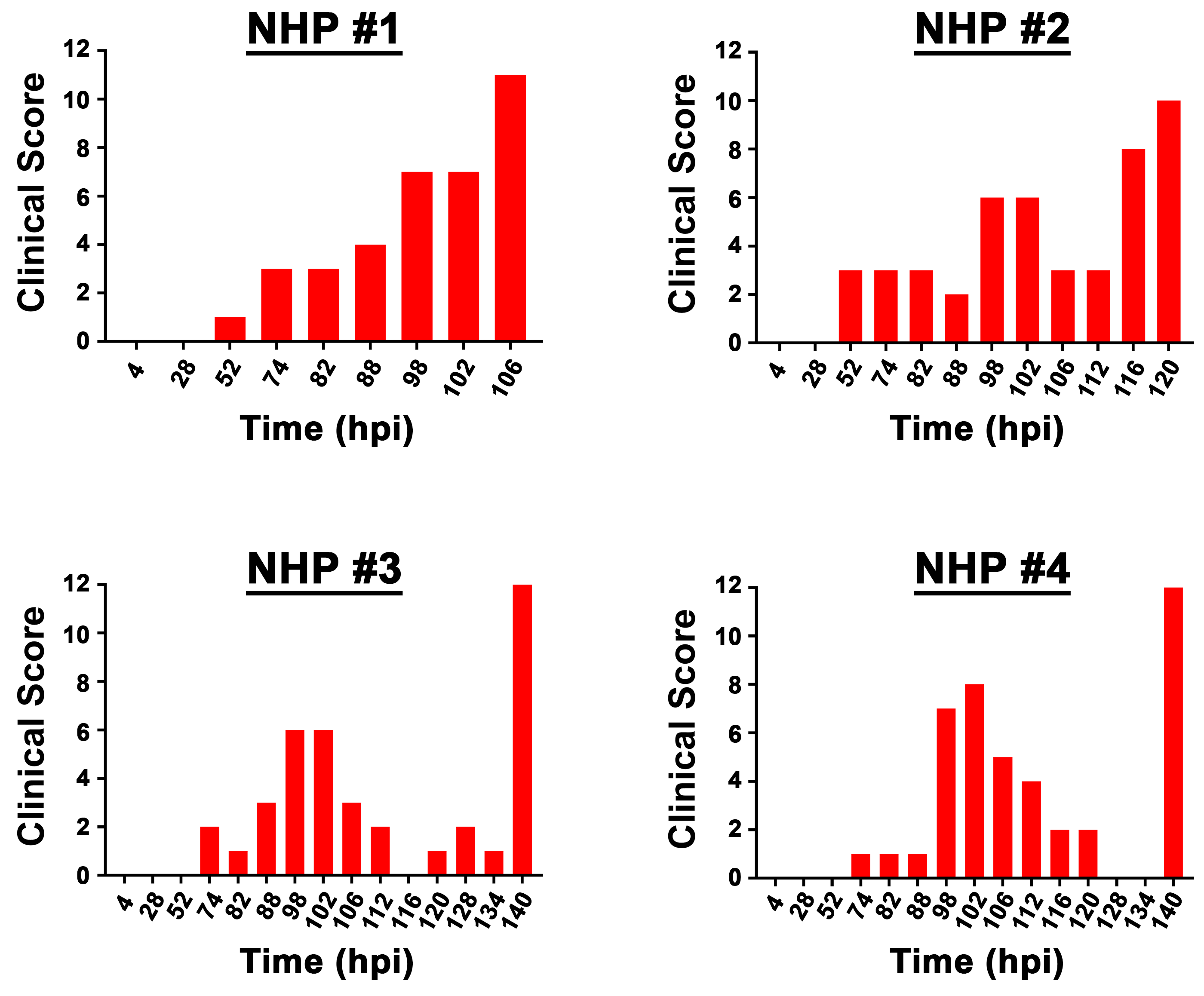

Supplement: S1 Fig — Following aerosol challenge all NHPs were monitored daily and NHPs with a total score ≥10 met the euthanasia criteria. (TIF) [file pntd.0009424.s001.tif]

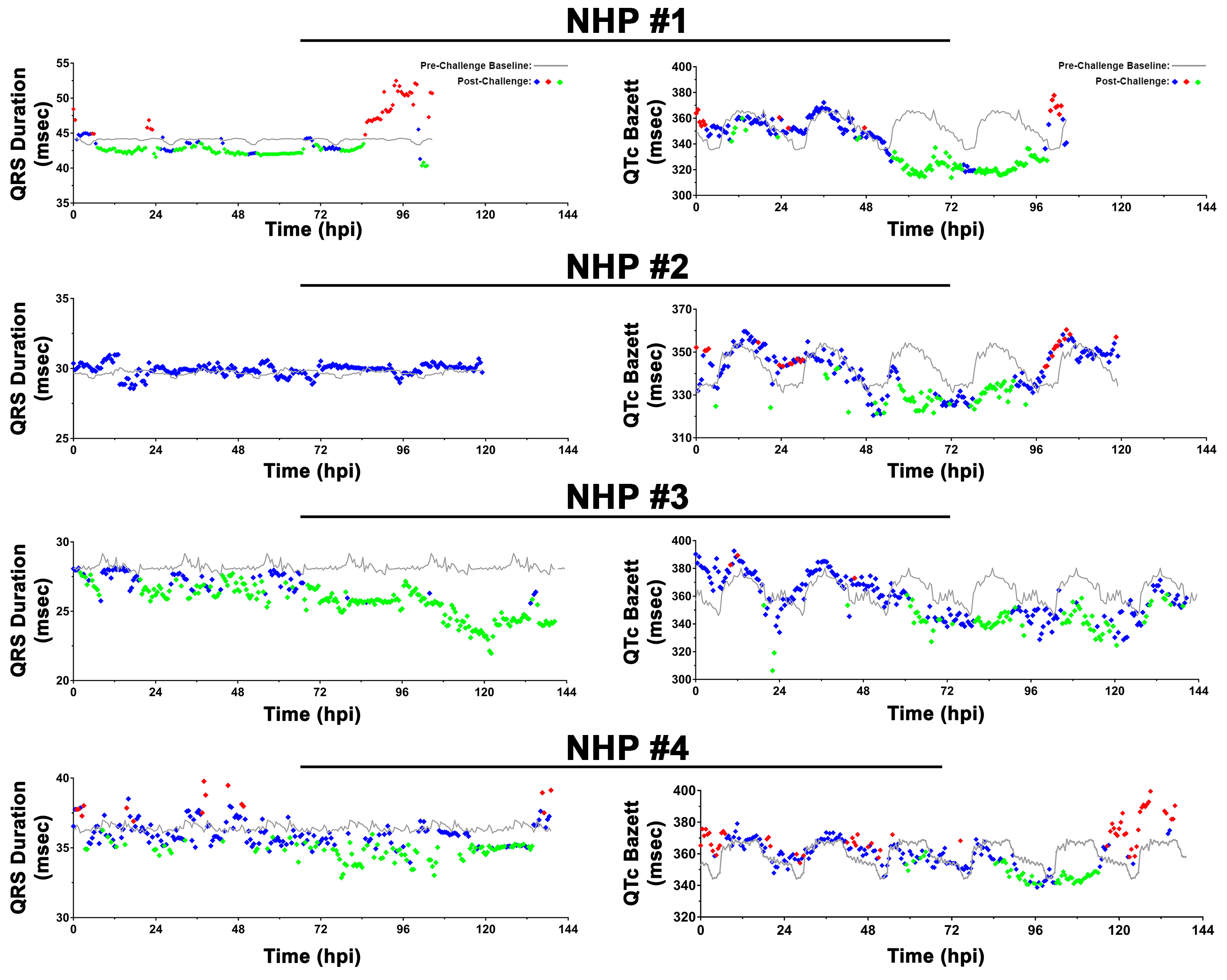

Supplement: S2 Fig — Pre-challenge baseline QRS duration and QTc Bazett are shown in grey (A). All NHPs were continuously monitored pre- and post-challenge. Pre-challenge baseline QRS duration and QTc Bazett were measured for five day/night cycles and a 0.5-hr interval baseline average was calculated by averaging raw data of five time-matched day or night time intervals. Forty-eight 0.5-hr interval averages were used to construct baselines for QRS duration and QTc Bazett for a day/night cycle and are shown as a grey line (A). Post-challenge values within ≤3 standard deviations (SD) are indicated with (), >3 SD above baseline are indicated with (), and >3 SD below baseline are indicated with (). (TIF) [file pntd.0009424.s002.tif]

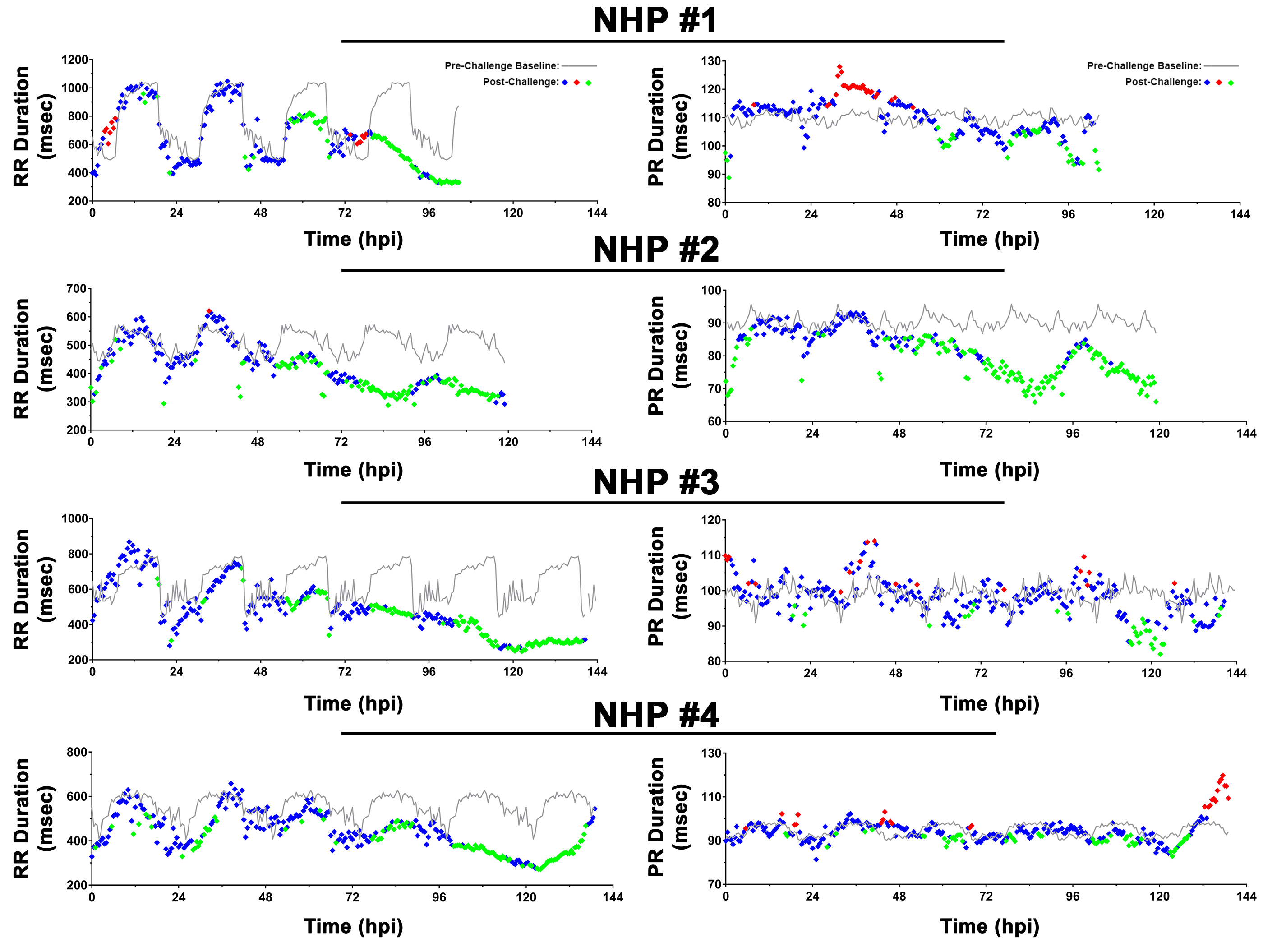

Supplement: S3 Fig — Pre-challenge baseline RR and PR duration are shown in grey (A). All NHPs were continuously monitored pre- and post-challenge. Pre-challenge baseline RR and PR duration were measured for five day/night cycles and a 0.5-hr interval baseline average was calculated by averaging raw data of five time-matched day or night time intervals. Forty-eight 0.5-hr interval averages were used to construct baselines for RR and PR duration for a day/night cycle and are shown as a grey line (A). Post-challenge values within ≤3 standard deviations (SD) are indicated with (), >3 SD above baseline are indicated with (), and >3 SD below baseline are indicated with (). (TIF) [file pntd.0009424.s003.tif]

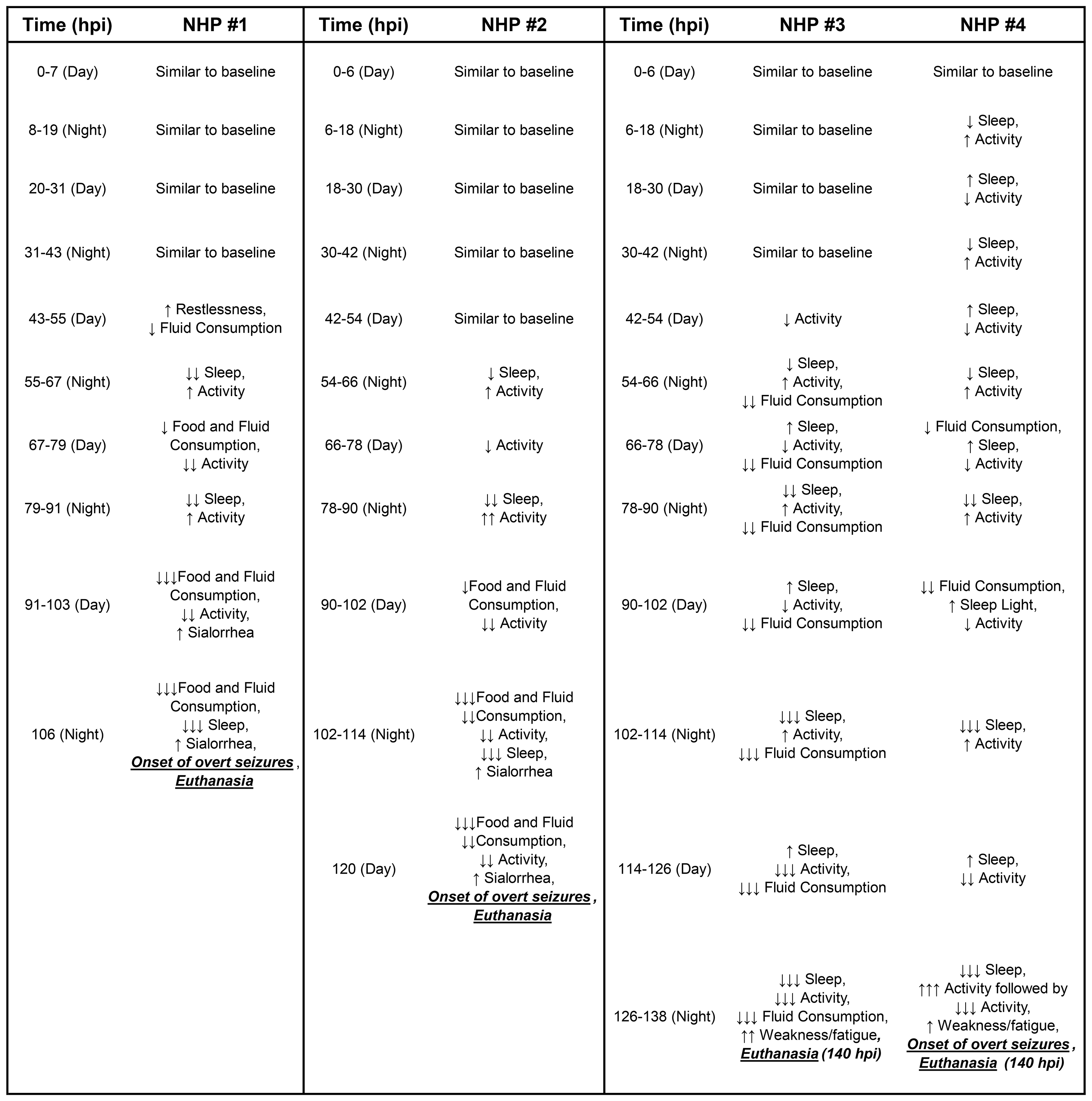

Supplement: S1 Table — NHP behavior comprised of food/fluid intake, sleep, activity, and onset of seizures were monitored pre- and post-EEEV challenge. ↓ = modest decline, ↓↓ = moderate decline, and ↓↓↓ = severe decline. ↑ = modest increase. (TIF) [file pntd.0009424.s004.tif]

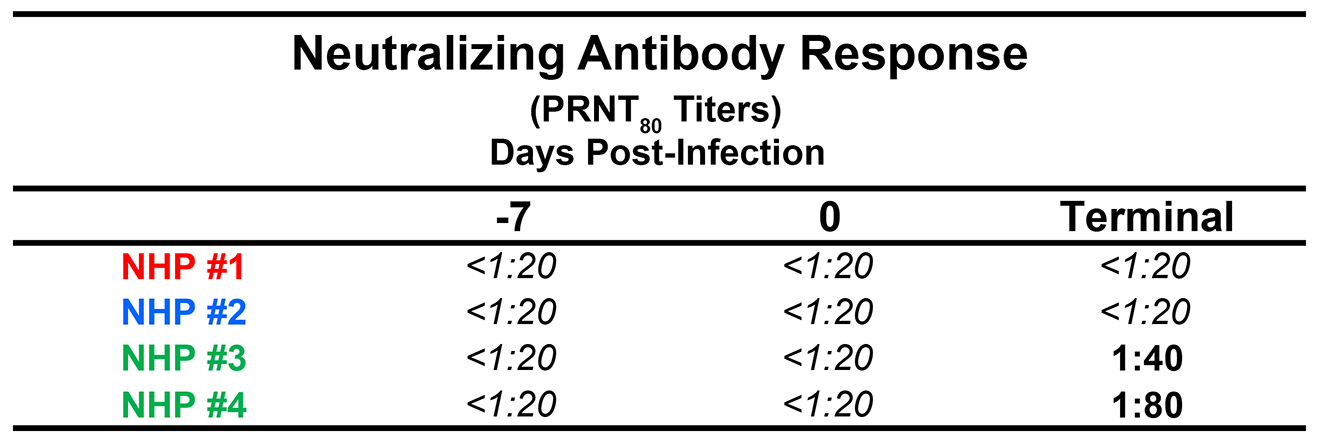

Supplement: S2 Table — Neutralizing antibody was measured via PRNT80 assay. The limit of detection in PRNT80 assay is indicated by italic font (<1:20). All samples were analyzed three times in the PRNT80 assay. (TIF) [file pntd.0009424.s005.tif]

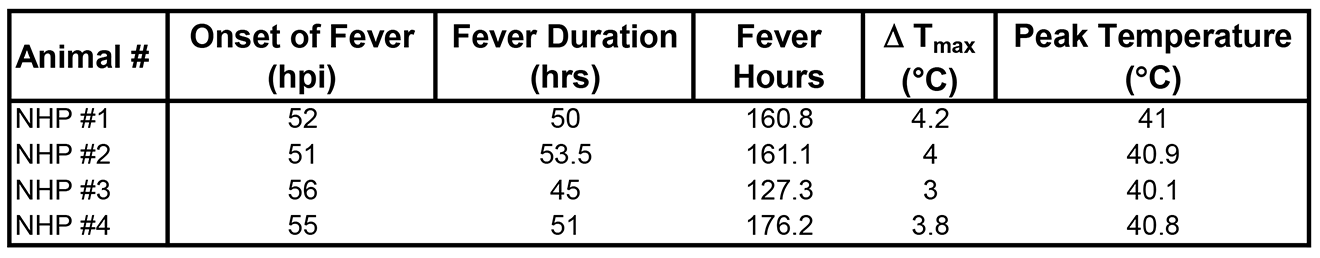

Supplement: S3 Table — Fever hours is calculated as the sum of the significant temperature elevations. ΔTmax = maximum change in temperature. (TIF) [file pntd.0009424.s006.tif]
