# Supplementary material for: The utilization of advance telemetry to investigate critical physiological parameters including electroencephalography in cynomolgus macaques following aerosol challenge with eastern equine encephalitis virus
Source: PLoS Negl Trop Dis. 2021 Jun 17;15(6):e0009424. doi: 10.1371/journal.pntd.0009424 (PMC8259972; doi:10.1371/journal.pntd.0009424)
Supplement: Attachment — Submitted filename: Responses to reviewers comments.pdf [file pntd.0009424.s007.pdf]

Methods

Reviewer #1

Comment #1: Is there any sequence or virological data relating to the viral stock used? This would be useful to include even if re-iterative.

Response #1: Thank you very much for the comment. The virus stock was deep sequenced and was identical to the published consensus sequence (Reference 16) (see lines 136-143).

Comment #2: Aerosol challenges often result in several log reductions in effective viral titre administered – can the authors comment on this and is there a dose effect in delivering different amounts of viral challenge with this system?

Response #2: Thank you very much for the comment. USAMRIID has extensive experience with the aerosol administration in both small and large animal models including non-human primates. The virus concentration in the nebulizer is 100 times higher than the target dose. We have added text for clarification (see lines 206-219).

Reviewer #2

Comment #1: One issue i have is the lack of justification for the use of NHPs to test the telemetry technology and this should be included in the text i.e why are NHP required? could mice/rats have been used instead?.

Response #1: Thank you very much for the comment. There are two reasons for the utilization of NHPs. One, the cynomolgus macaques can recapitulate many aspect for human disease (See lines 125-129). Two, the device telemetry device size are large and are not appropriate for small animal models.

Comment #2: As a final thought on experimental design, I wasn't entirely sure why the authors infected the NHPs via the aerosol route, given that the primary route of infection is through the skin during a mosquito blood meal. I may have missed it, but an additional sentence to justify this would improve the rationale of the manuscript.

Response #2: Thank you very much for the comment. The subcutaneous route does not produce uniform lethal disease (see reference below). This was the first detailed telemetry study to investigate many critical physiological parameters in NHPs to mirror fatal human disease progression. Consequently, the study was designed to produce uniform severe disease in NHPs via an established aerosol route (See references 14-15).

Smith DR, Schmaljohn CS, Badger C, Ostrowski K, Zeng X, Grimes SD, Rayner JO. Comparative pathology study of Venezuelan, eastern, and western equine encephalitis viruses in non-human primates. Antiviral Res. 2020 Oct;182:104875.

Reviewer #3

Comment #1: However, the main new method presented here (i.e. the telemetry system) is poorly described and only brand mark and analytic performance were provided. Personally, I wonder if the very high cost of such a system deserved to be provided. Even if the specific method of aerosol challenge is already well described, the time cost and method used to implement the animals with the telemetry device should be provided here.

Response #1: Thank you very much for the comment. We have added a detailed section on the telemetry devices and data collection (See lines 165-187). In regards to the high cost issue, the telemetry system is cost effective. However, the implantation of devices in NHPs in complex. This is comprised of surgeon skill and experience, availability of experienced surgeons, techniques to implant devices, and NHP species. We were extraordinarily fortunate to have the research funds to collaborated with Covance and DSI to develop a successful streamlined process to obtain implanted animals. Currently, NHPs with desired implanted devices can be ordered by any institution, Covance/DSI will perform the surgeries to implant devices, ensure functionality, and will deliver the NHPs. However, the discussion of telemetry implantation challenges and acquisition of NHPs is beyond the scope of the current manuscript.

Comment #2: As an example, how long time is needed in order the animals recovered from the device implantation, is there a difference or not between animals of 3kg or 9 kg as indicated. I imagine the female and male differences as the sexual dysmorphism is high in this macaque species. Could you please provide this information in the table A) within figure 2.

Response #2: Thank you very much for the comment. The weight of NHPs is now provided in Figure 2. We collaborated with Covance and DSI to implant the animals at their facilities and received the animals 4 weeks after the implantation. The weight range was due to the size of the implanted devices and was determined by experienced veterinarians at Covance and DSI. According to Covance veterinarians the NHPs recovered within 1-2 days post-surgery and the healing of the surgical incision sites was ~2 weeks. We have added a detailed section on the telemetry devices and data collection (See lines 165-187).

Comment #3: At least, references to the device provider reports or vet article describing these procedures might be of interest for the non-specialized reader to highlight the work needed here. Could the device be re-used ? are all animals infected at the same time or successively ?

Response #3: Thank you very much for the comment. The procedures to implant the devices were non-routine and/or new. Our DSI/Covance collaborators are preparing manuscripts detailing the methods. The M series devices are not reusable. Also due to biosafety issues, we do not recommend any re-usable devices. Lastly, one animal was aerosol exposed at time (See line 219).

Results

Reviewer #1

Comment #1: For small numbers of animals, it is instructive to relate the limited but valuable virological data generated (supplementary figure 2) directly back to the clinical observations. Were earlier serum or plasma samples taken that would confirm on-set or presence of a viraemia in this model. Were there any cytokine changes noted? Some reference to these virological and immunological variables would be instructive.

Response #1: Thank you very much for the comment. To obtain any virological or immunological data from the in-life part of the NHP study would require the use of anesthesia. Considerable after effects of anesthesia were noted during the establishment of baseline and animal physical. These after effects led to significant confounding of the telemetry data for all parameters. Consequently, we decided not to characterize the viremia or immune responses during the in-life portion of the study.

Comment #2: It would be extremely valuable to relate the infectious virus titre data generated more specifically to the clinical observations. For example, NHP#1 is the only animal to have a detectable viraemia in serum and plasma (approx. 3.5 log 10Pfu). The other three are undetectable – this on the face of it would seem a significant observation, at least worthy of a brief comment. NHP#3 has 4 logs of virus in the heart but the other three are undetectable – how does this relate to heart output measures? - again no direct comment by the authors. Linking these observations would hugely enrich the manuscript.

Response #2: Thank you very much for the comment. We have added a paragraph addressing the comments in the discussion section (See lines 669-687). The presence of virus in the heart of one NHP will be addressed in a follow up manuscript detailing the pathology in these NHPs.

Comment #3: Infectious virus detected in the olfactory bulb in all 4 and in the brain is at least worthy of a comment on the route of virus trafficking to the brain via an aerosol administration – is there any pathology data that would inform these events? Perhaps not the focus of this study and beyond current scope but would be very interesting follow-up study if the materials existed or were collected.

Response #3: Thank you very much for the comment. We have added a paragraph addressing the comments in the discussion section (See lines 669-687). We did perform

a detailed pathology study to examine the tissues from each animal and will submit the data as a separate manuscript.

Comment #4: Are there any PCR data for this model that could be generated/included, - virus-specific cell-associated RNA quantification would be a useful additional biomarker to relate across to the infectious virus data.

Response #4: Thank you very much for the comment. Our data shows the presence of infectious virus in the brain at orders of magnitude higher than in the periphery. Thus strongly suggesting that the infection is predominantly limited to the brain. In addition, the PCR would only pick up viral RNA and not infectious virus. We focused our efforts to investigate the presence of infectious virus as it is the most relevant.

Comment #5: Hence, it would seem highly relevant to relate these limited, but nonetheless informative virological findings back/across to the physiological data. To enable this I would recommend moving Supp Fig 2 into the main body of the manuscript and integrating these data into the descriptions of the results and work into the discussion as appropriate.

Response #5: Thank you very much for the comment. The figure has been moved the main text (See Figure 3).

<u>**Reviewer #2**</u>

Comment #1: Section "EEEV challenge study" - It would improve the entire results section if a brief description of clinical signs was added here to help the reader interpret the results.

Response #1: Thank you very much for the comment. A description for clinical signs is in lines 220-233 and 274-278.

Comment #2: Supp. figure 1 is very confusing, the sampling is poorly explained, i.e. why were the animals sampled different number of times depending on the dpi? I think it would be more appropriate to display that as hours post infection, in line with the other figures in the manuscript.

Response #2: Thank you very much for the comment. The figure has been updated (see Supp. Figure 1).

Comment #3: An attempt should also be made to discuss the bi-phasic disease profile in NHP 3 and 4, is it expected that NHP 4 would have recovered to have 0 clinical score before euthanasia? or is this a limitation of the scoring system itself?

Response #3: Thank you very much for the comment. This is a not a limitation of the scoring system but rather the EEEV infection. The animals were not scoring and yet

progressed rapidly to terminal phase. A paragraph has been added to address the issue (See lines 591-598 and 688-700).

Comment #4: "Detection of Infectious EEEV in Various Tissues" - Although interesting, due to the low N number and spread of results (also the difference in endpoints), the biological relevance of the tissue tropism is difficult to interpret. I would like to see the relevance and limitations of the viral loads and tissue tropism discussed in the discussion section, alternatively, as this is supplementary, perhaps consider making this section more succinct and amalgamating it with "EEEV Challenge Study" as tissue tropism isn't a strong focus of this manuscript.

Response #4: Thank you very much for the comment. We have added a paragraph, and reorganized and combined the suggested sections (see lines 273-302, see lines 669-687).

Comment #5:"Neutralizing Antibody Response" - Figure 2C would be more appropriate as a supplemental figure, It is important prior to infection to demonstrate the NHPs did not have neutralising Ab, however more work is required to characterise the adaptive immune response, especially as the time post infection is too early for class switching, high affinity neutralizing antibodies. Additionally, the number of repeats for the PRNT assay should be stated in the legend clearly, to ensure this isn't an artefact.

Response #5: Thank you very much for the comment. We have reorganized the figure and moved the neutralizing antibody table in the supplemental material (See Supp. Table 2). Additional text has been added to the Materials and Methods section and the figure legend (See lines 264-265 and 1391-1392).

Comment #6:"Temperature." - This is where this manuscript really starts to assess the telemetry technology, where i believe it's strengths lie. I have a few questions to be clarified: The baseline for 3A (this is relevant for figure 4-8) is confusing, is the baseline taken for the whole 144 hours pre infection? or is it repeating measurements over a 24 hours? because the grey line looks remarkably consistent for a parameter such as temperature. to Avoid confusion and increase transparency, figure 3A should be presented like 3B i.e. with the X-axis starting at -96hpi and finishing at 144hpi

"respiration" - same as above, interesting data which would be improved by tweaking the presentation of the results of A to mirror that of B.

"Activity - blood pressure" - Same as above, consider changing presentation

"ECG" -Caveat being, this isn't my area of expertise, but it looks well presented to me.

Response #6: Thank you very much for the comment. To clarify how the baseline for each parameter was determined we have added sections to Materials and Methods Section and figure legend (see lines 188-205, 274-278, 1227-1313, and 1361-1380).

Conclusions

Reviewer #1:

Comment #1: With any virological NHP challenge study, however, there will be inevitable variations in the replication dynamics and host response to the virus. The authors could make a general point as to why Chinese cynomolgus macaques are the model of choice here and perhaps compare to other cynomolgus macaque species (eg Mauritian or Indonesian), as to why select one species over another. Would different outcomes be anticipated in such circumstances? Integrating combined physiological outcomes with the virological data presented in the discussion would improve the paper. Public health relevance is well stated and teh authors describe how this model relates to this.

Response #1: Thank you very much for the comment. The utilization of Chinese cynomolgus is institutional knowledge obtained by performing of numerous NHP studies with large group size >10. Multiple studies performed with Mauritian cynomolgus yielded remarkably uniform results, whereas macaques from different sources yielded the expected variation. The most likely explanation for the observed difference in Mauritian cynomolgus is lower genetic variation than the Chinese sourced macaques. Other publications from our institution will address this issue in the near future.

Reviewer #2:

Comment #1: The conclusions of this manuscript are clearly stated and nicely put into context of the work that has previously been undertaken. Moreover, a satisfactory job has been made to discuss the findings in the context of how telemetry could be useful for future studies. One minor issue is that portions of interesting data are presented as if this is a preview for a later, more in-depth study, i Would like to see statements such as "The underlying cause/s of ECG abnormalities are currently under investigation", and "sub-lethal EEEV dose remains to be seen and will be investigated in future studies" removed or reworded.

Response #1: Thank you very much for the comment. We have deleted or reworded the suggested lines (see lines 594-598, 629-630, 668, and 717-721).

Reviewer #3:

Comment #1: In addition, could the author may indicate how they investigate the underlying mechanisms as stated line 458-459 and 486-487 (heart and brain structure evaluation and/or virus research). Will this only be done with the materials from the described 4 animals or this will involve historical samples (and which type of samples) from previous study of the very same model? Even, if out of the main scope of this

article, how the author will take in account the variability of the death timing (here between 4 and 6 days)?

Response #1: Thank you very much for the comment. We have performed a detailed pathology study of all organs and tissues encompassing microscopic lesions, presence of viral RNA and proteins, virus tropism, and virus localization within cells via electron microscopy to elucidate the underlying mechanism. In addition, despite the difference in time of euthanasia, the underlying mechanism is remarkably consistent. A manuscript is under preparation and can be shared with the reviewer upon request.

Editorial and Data Presentation Modifications?

Reviewer #1:

Comment #1: Move supplementary figure 2 into the bulk of the manuscript and integrate these observations into the descriptions of the physiological data/measures.

Response #1: Thank you very much for the comment. The figure has been moved and incorporated to the main text.

Reviewer #2:

Comment #1: Figure 1 - indicate on figure where data is collected i.e. if continuous, state that in the figure.
Figure 2 - state number of technical repeats in PRNT assay in the figure legend
Figure 3 through 8 - be clear how the baseline was calculated in A, consider changing the presentation of "A" to match "B"
Figure 9 - no comment
Figure 10 through 11 - not my area of expertise but it is easy to interpret
Figure 13 - again, confusing how and when the baseline is calculated? are they matched to the same time on different days? clarify in the figure legend

Supp.1 - discuss limitations of clinical score in the text, is it expected that they would recover immediately prior to euthanasia?
Supp.2 - consider removing CSF in this figure, the incomplete data set is confusing
Supp. 3 through 4 - consider changing style to make the baseline clearer

Response #1: Thank you very much for the comment. Extensive edits have been made to throughout the manuscript to address the edits (see lines 188-205, 264-265, 274-278, 591-598, 688-700, 1227-1313, and 1361-1380).

Reviewer #3:

Comment #1:

Minor remarks:
1) The term Chinese origin for cynomolgus is more a producer brand as the natural range of Macaca fascicularis do not reach China mainland. Thus, an indication of the genetic background of these animals might be of interest as at least 3 main groups are well known: from 1) Vietnam and Asia continental area to 2) Indonesia archipelago and finally 3) in Philippian archipelago. And this, when we take not in account the very specific case of the Mauritius sub-group that is largely used in biomedical research.

Response #1: Thank you very much for the comment. We have stated the country of origin on the birth certificate. Also, the utilization of Chinese cynomolgus is institutional knowledge obtained by performing of numerous NHP studies with large group size >10. Multiple studies performed with Mauritian cynomolgus yielded remarkably uniform results, whereas macaques from different sources yielded expected variation. The most likely explanation for the observed difference in Mauritian cynomolgus is lower genetic variation than the Chinese sourced macaques. Other publications from our institution will address this issue in the near future.

Comment #2: please provide a reference for the aerosol exposition method and viral titer evaluation for the exposition in the result lines.

Response #2: Thank you very much for the comment. We have added a section in the Materials and Methods section (see lines 206-219).

Comment #3: Could you explain (or have an hypothesis) the major modification for the NHP#1 at -16 pre-challenge in figure 10 ? There were also some blue in the baseline for NHP#4

Response #3: Thank you very much for the comment. This is due to human activity in the room when the NHPs were moved into containment and devices were turned on as well as the day prior to challenge. We have added sentences for clarity (see lines 435-437).

Summary and General Comments

Reviewer #1:

Comment #1: Trefry and colleagues describe the use of advanced telemetry techniques to monitor a range of physiological read-outs in a Chinese cynomolgus macaque model of Eastern equine encephalitis virus following aerosol administration. This a very comprehensive description of the range of physiological measurements that can be undertaken the specialist facility at USAMRIID. The large number of Figures summarising and describing these data reflect the range of measures that have been undertaken. Development and refinement of models to investigate the possible outcomes of virus infection in understanding the pathogenesis of the virus and to develop suitable models to evaluate intervention strategies are hugely valuable.

As outlined in Results the infectious virus load data needs to brought into the paper more.

Response #1: Thank you very much for the comment. We have moved the infectious virus figure into the main manuscript (See figure 3).

Comment #2: Better integration of these virological observations to the clinical/physiological measures taken would enhance this paper enormously. Do the authors intend to follow up on brain pathology? This would seem an obvious next step and to maximise the resources of this hugely valuable study which is beyond the scope of most institutions.

Response #2: Thank you very much for the comment. We have added text to the discussion (see lines 669-687). We have performed a detailed pathology study of all organs and tissues encompassing microscopic lesions, presence of viral RNA and proteins, virus tropism, and virus localization within cells via electron microscopy to elucidate the underlying mechanism. A manuscript is under preparation and can be shared with the reviewer upon request.

Reviewer #2:

Comment #1: There is a few outstanding issues with the presentation of this manuscript which can be easily improved with extra clarification in the text, including more robust justification for using NHPs, changes to how baselines are presented and some amalgamation of less relevant data sets i.e. antibody response and tissue tropism. Generally, a good attempt has been made to put this data into the broader context of the literature, however, much of the manuscript feels incremental, and i would have loved to see the inclusion of work relating to the infection of the autonomic nervous system, the cardiac event of NHP1 and the mechanisms of traumatic brain injury.

Response #1: Thank you very much for the comment. To clarify how the baseline for each parameter was determined we have added sections to Materials and Methods Section and figure legend (see lines 188-205, 274-278, 1227-1313, and 1361-1380). We have performed a detailed pathology study of all organs and tissues encompassing microscopic lesions, presence of viral RNA and proteins, virus tropism, and virus localization within cells via electron microscopy to elucidate the underlying mechanism. A manuscript is under preparation and can be shared with the reviewer upon request.
